# Moderate alcohol drinking in pregnancy increases risk for children's persistent conduct problems: causal effects in a Mendelian randomisation study

**DOI:** 10.1111/jcpp.12486

**Published:** 2015-11-21

**Authors:** Joseph Murray, Stephen Burgess, Luisa Zuccolo, Matthew Hickman, Ron Gray, Sarah J. Lewis

**Affiliations:** ^1^ Department of PsychiatryUniversity of CambridgeCambridgeUK; ^2^ Department of Public Health and Primary CareUniversity of CambridgeCambridgeUK; ^3^ School of Social and Community MedicineUniversity of BristolBristolUK; ^4^ MRC Integrative Epidemiology UnitUniversity of BristolBristolUK; ^5^ National Perinatal Epidemiology UnitNuffield Department of Population HealthUniversity of OxfordOxfordUK

**Keywords:** Foetal alcohol effects, conduct disorder, longitudinal study, mendelian randomization analysis, ALSPAC

## Abstract

**Background:**

Heavy alcohol use during pregnancy can cause considerable developmental problems for children, but effects of light‐moderate drinking are uncertain. This study examined possible effects of moderate drinking in pregnancy on children's conduct problems using a Mendelian randomisation design to improve causal inference.

**Methods:**

A prospective cohort study (ALSPAC) followed children from their mother's pregnancy to age 13 years. Analyses were based on 3,544 children whose mothers self‐reported either not drinking alcohol during pregnancy or drinking up to six units per week without binge drinking. Children's conduct problem trajectories were classified as low risk, childhood‐limited, adolescence‐onset or early‐onset‐persistent, using six repeated measures of the Strengths and Difficulties Questionnaire between ages 4–13 years. Variants of alcohol‐metabolising genes in children were used to create an instrumental variable for Mendelian randomisation analysis.

**Results:**

Children's genotype scores were associated with early‐onset‐persistent conduct problems (OR = 1.29, 95% CI = 1.04–1.60, *p *=* *.020) if mothers drank moderately in pregnancy, but not if mothers abstained from drinking (OR = 0.94, CI = 0.72–1.25, *p *=* *.688). Children's genotype scores did not predict childhood‐limited or adolescence‐onset conduct problems.

**Conclusions:**

This quasi‐experimental study suggests that moderate alcohol drinking in pregnancy contributes to increased risk for children's early‐onset‐persistent conduct problems, but not childhood‐limited or adolescence‐onset conduct problems.

## Introduction

Conduct problems are characterised by antisocial behaviours, such as stealing, lying, fighting and aggressive outbursts, which can cause considerable difficulties for children and families, and result in substantial costs to society (Scott, Knapp, Henderson, & Maughan, 2001). A minority of children show conduct problems in early childhood that persist into adolescence, and predict risk for adult psychiatric disorder, substance misuse and addiction, poor physical health, domestic violence, crime, financial problems and unemployment (Heron et al., 2013; Kretschmer et al., 2014; Odgers et al., 2007; Stringaris, Lewis, & Maughan, 2014). Prominent theories suggest that early‐onset‐persistent conduct problems have different origins to more transient or less serious problem behaviours, and highlight the role of neurodevelopmental factors in foetal development and early childhood (Moffitt, 1993; Moffitt & Caspi, 2001; Raine, 2002). However, identifying true causes of conduct problem behaviour, as opposed to mere correlates, is a major challenge and leading scholars have argued that ‘antisocial behaviour research is stuck in the risk‐factor stage’ (Moffitt & Caspi, 2006, p. 109).

Prenatal exposure to alcohol is one possible cause among many bio‐psych‐social factors contributing to children's conduct problems. Blood‐alcohol concentrations in the foetus reach similar levels to maternal blood‐alcohol concentrations within about 2 hr of maternal drinking, and persist longer in the foetus than in the mother because of slower foetal metabolism of alcohol and recycling of alcohol in amniotic fluids (Burd, Blair, & Dropps, 2012). Since the seminal reports recognising foetal alcohol syndrome over four decades ago (Jones & Smith, 1973; Lemoine, Harousseau, Borteryu, & Menuet, 1968), human and animal studies have consistently shown adverse consequences of heavy drinking in pregnancy for offspring's physical, neurological and behavioural development (Jacobson & Jacobson, 2002; Mattson, Schoenfeld, & Riley, 2001; Mukherjee, Hollins, & Turk, 2006). However, studies examining children's conduct problems after light‐moderate maternal drinking in pregnancy (maximum of six units of alcohol per week, and small amounts consumed on each drinking occasion) have conflicting results (D'Onofrio et al., 2007; Kelly et al., 2009, 2010; Larkby, Goldschmidt, Hanusa, & Day, 2011; O'Leary et al., 2010; Robinson et al., 2010; Skogerbø et al., 2013; Sood et al., 2001).

A key challenge to testing for causal effects of foetal alcohol exposure is that drinking and nondrinking women differ in many ways. Women who drink in moderation during pregnancy are, on average, older and better educated, and have higher incomes and better mental health than nondrinking women, which may confound any association (Falgreen Eriksen et al., 2012; Robinson et al., 2010; Stranges et al., 2006). Mendelian randomisation studies (Davey Smith & Ebrahim, 2003; Lewis, Relton, Zammit, & Davey Smith, 2013; Pingault, Cecil, Murray, Munafò, & Viding, in press) use genetic instrumental variables to reduce bias from confounding, and have shown possible effects of moderate drinking in pregnancy on children's IQ (Lewis et al., 2012) and school performance (Zuccolo et al., 2013). However, to our knowledge, no previous study has used this method to examine effects on children's behaviour. The current study investigates effects of maternal moderate drinking in pregnancy on children's conduct problems, and is novel both in using a Mendelian randomisation design and examining different longitudinal trajectories of children's conduct problems as outcomes.

## Methods

### The logic of the Mendelian randomisation study design

Mendelian randomisation studies use naturally occurring genetic variants to assess causal effects of environmental risk factors on outcomes (Davey Smith & Ebrahim, 2003; Lewis et al., 2013; Pingault et al., in press). The strength of the design stems from the fact that genotypes are randomly inherited at conception, following Mendel's second law of ‘independent assortment’. Mendelian randomisation studies examine whether genetic variants that contribute to variation in the environmental risk factor also predict the study outcome. Assuming that the genetic variants are not associated with confounding variables (following Mendel's law), and only influence the outcome via the risk factor, associations between the genetic variants and the outcome provide evidence of causal effects of the environmental risk factor. Mendelian randomisation studies can therefore move research beyond documentation of risk factors towards identification of causes.

**Figure 1:**
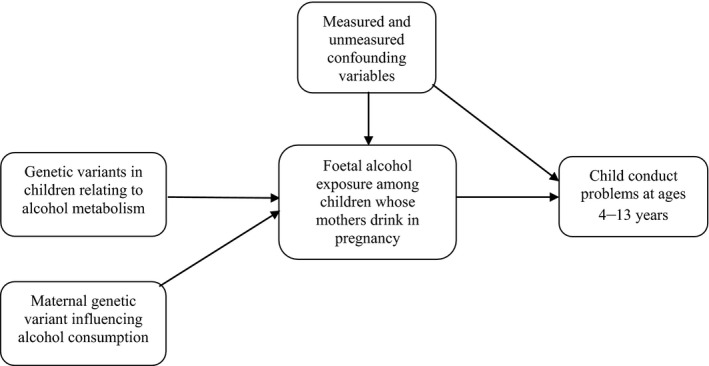
Mendelian randomisation design for testing the effects of maternal drinking in pregnancy on children's conduct problems

Levels of foetal alcohol exposure vary both according to how much pregnant mothers drink and the extent to which a foetus metabolises that alcohol. The main tests in the current study use child genes affecting foetal alcohol metabolism to examine the effects of foetal alcohol exposure on child conduct problems (see Figure 1). Exploratory analyses also examine whether a maternal genetic variant affecting maternal alcohol consumption (Zuccolo et al., 2009) predicts children's conduct problems.

Alcohol is primarily metabolised by a group of five alcohol dehydrogenase (ADH) enzymes (ADH1A, ADH1B, ADH1C, ADH4, ADH7). Peak alcohol levels are higher and persist for longer among slow metabolisers compared to fast metabolisers (Birley et al., 2009). Variations in genes that encode these enzymes lead to differences in the extent to which foetuses metabolise alcohol consumed by their mothers (Birley et al., 2009, 2008). Hence, by comparing outcomes for children with different ADH alleles (variants of the same gene affecting alcohol metabolism), we test whether variations in foetal alcohol exposure affects children's conduct problems.

Importantly, children's genetic variations affecting alcohol metabolism will cause different foetal alcohol levels only if pregnant mothers actually drink alcohol. Therefore, if foetal alcohol exposure does influence children's behaviour, the relevant genetic variants should be associated with conduct problems among children of drinking mothers, but this should *not* be true among children of nondrinking mothers. It is the effects of moderate drinking that are least certain and most debated regarding public health. By restricting analyses to mothers who did not drink heavily, the comparisons in this study indicate whether small differences in foetal alcohol exposure among children of moderately drinking mothers influence children's conduct problems.

### Participants

The Avon Longitudinal Study of Parents and Children (ALSPAC) is a population‐based prospective study investigating environmental and other factors affecting the health and development of children. The study methods have been previously described in detail (Boyd et al., 2013; Fraser et al., 2013) and the study website contains information on all the data that is available through a fully searchable data dictionary (http://www.bris.ac.uk/alspac/researchers/data-access/data-dictionary/). In brief, ALSPAC recruited 14,541 pregnant women resident in Avon, Britain with expected dates of delivery 1 April 1991 to 31 December 1992; and, from age 7, continued to recruit children born in that area at that time until age 18. Detailed information was obtained from the mother throughout pregnancy and information on both the mother and child has been collected at regular intervals, and is ongoing. Ethical approval came from the ALSPAC Law and Ethics Committee (IRB 00003312) and four Local Research Ethics Committees. After complete description of the study to the subjects, written informed consent was obtained.

**Figure 2:**
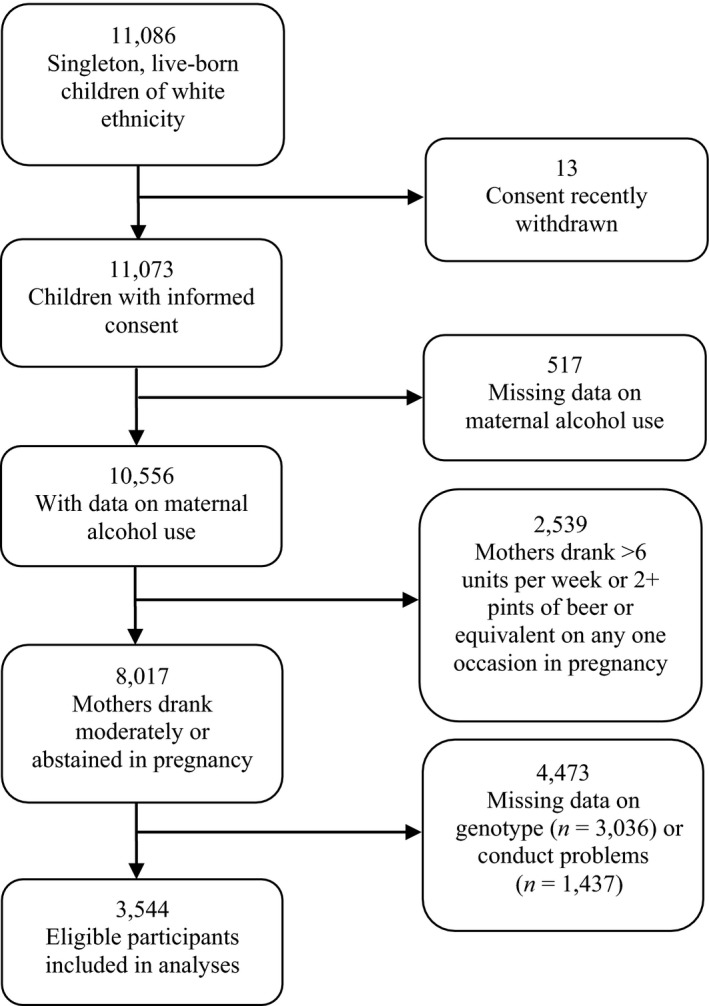
Flow chart of sample included in analyses

The current study started with 11,086 live‐born singleton children of white ethnicity. Figure 2 shows that 8,017 of those children's mothers reported moderate drinking or abstinence during pregnancy, for which 3,544 had complete data and were included in the analyses. Comparing children included in the analyses with children otherwise eligible, but excluded because of missing genotype or conduct problem data, the two groups differed (all *p *<* *.001) in terms of maternal age (mean = 29.3 vs. 27.6 years), smoking in pregnancy (13.8% vs. 24.6%), depression in pregnancy (8.0% vs. 13.5%), manual social class (13.8% vs. 21.8%) and low education (53.5% vs. 66.1%). However, there was no difference in the distribution of our key variable (child genetic risk score – see below) between complete and missing cases.

## Measures

### Alcohol use

Maternal alcohol use was measured in self‐report questionnaires at 18 and 32 weeks gestation. At 18 weeks, women reported their average amount and frequency of alcohol consumption in the first trimester, and regarding the previous 2 weeks or at the time when they first felt the baby move. At about 32 weeks gestation, women completed another questionnaire in which they were asked about their average weekday and weekend alcohol consumption. Women who reported drinking any amount of alcohol at any stage of pregnancy were classified as drinking during pregnancy. Women for whom the 32 week questionnaire was missing were not excluded from analyses, but coded according to their drinking status measured at 18 weeks.

There are no standardised definitions of moderate and heavy alcohol use in pregnancy. We defined moderate drinking using questionnaire response categories that indicated consuming >0–6 units of alcohol per week at any time in pregnancy and not drinking >6 units per week or two pints of beer or equivalent on a single occasion at any time in pregnancy. This is similar to the definition of light‐moderate drinking used by the British Medical Association and Department of Health (British Medical Association, 2007, p. iv).

### Conduct problem trajectories

Maternal reports of child conduct problems were collected when children were aged 4, 7, 8, 10, 12 and 13 years using the Strengths and Difficulties Questionnaire (SDQ), a widely used screening instrument with well‐established reliability and validity (Goodman, 2001). Using cases with 4–6 SDQ scores between ages 4–13, four trajectories of conduct problems were previously identified in growth mixture models (Barker & Maughan, 2009; Heron et al., 2013; Kretschmer et al., 2014): a low‐risk class, maintaining low levels of conduct problems throughout the study; a childhood‐limited class, initiating conduct problems in early childhood but desisting to low levels by age 13; an adolescence‐onset class, starting at low levels of conduct problems but increasing by age 13, and an early‐onset‐persistent group, initiating conduct problems early in childhood and persisting into early teens.

### Potential confounders

Maternal age, smoking in pregnancy, antenatal depression, social class and education were measured in questionnaires administered to the mother in pregnancy, including the Edinburgh Postnatal Depression Scale (Cox, Holden, & Sagovsky, 1987) administered at 18 and 32 weeks gestation. We used a cut‐off point of ≥13 to identify women with depressed mood for our analyses. Mother's social class was based on occupation and determined according to the 1991 British Office of Population Statistics classification, and dichotomised as manual/lower versus higher. Mother's education was dichotomised as having up to Ordinary Level (O‐level) qualifications or equivalent versus higher. The O‐level was an exam‐based qualification used until 1988 in the United Kingdom for students aged 14–16 years. Ethnicity was available from self‐report or had been imputed from five genetic ancestry‐informative markers (Zuccolo et al., 2009). Women and children of white‐European origin only were included in analyses to avoid population stratification, as many polymorphisms in ADH genes differ markedly across different populations (Osier et al., 2002) and patterns of alcohol drinking are culturally dependent.

### IQ

Since we have previously shown that the genetic variants used in this study are associated with IQ in children of mothers who drank in moderation in pregnancy (Lewis et al., 2012), we examined whether foetal alcohol exposure predicted child conduct problems independently of child IQ. IQ was measured during a clinic visit of children at age 8 using a shortened version (Joinson et al., 2007) of the Wechsler Intelligence Scale for Children (WISC‐III, Wechsler, Golombok, & Rust, 1992) from which an overall age‐adjusted total score was derived.

### Genetic variants

Foetal exposure to alcohol is likely to be affected by multiple genetic variants. Four single nucleotide polymorphisms (SNPs) in three ADH‐related genes of the child (*ADH1A* rs2866151, rs975833, *AHD1B* rs4147536, and *ADH7* rs284779) were selected for use as instrumental variables. These four variants are in alcohol‐metabolising genes (Birley et al., 2009, 2008) and were a minimum set of markers which predicted children's IQ among children of drinking mothers, taking into account linkage disequilibrium (Lewis et al., 2012). Each child could carry zero, one or two rare alleles at each locus. Most children (3,354 of 3,544 = 94.6%) had a total of two, three or four ‘risk’ alleles. Seven children had no risk alleles, 162 children had one risk allele, 21 had five risk alleles and none had more than five risk alleles. Children were grouped as having ≤2, 3, or ≥4 risk alleles for analyses.

In secondary, exploratory analyses, we also examined the relationship between children's conduct problems and the maternal genotype *ADH1B* wildtype rs1229984. Rs1229984 is a nonsynonymous variant thought to have a functional role in alcohol metabolism. In vitro studies showed up to 40 times higher enzymatic activity in liver samples from carriers versus noncarriers of the A allele (Yin, Bosron, Magnes, & Li, 1984), resulting in faster accumulation of plasma acetaldehyde. There is strong evidence in ALSPAC that this variant predicts greater maternal drinking in pregnancy compared to the rare allele, and that it is unassociated with other maternal characteristics, including education, socioeconomic status and age (Zuccolo et al., 2009). For instance, mothers with the *ADH1B* wildtype were more likely (OR = 1.55; CI = 1.24–1.93, *p *<* *.001) than mothers with one or more copies of the rare allele to drink any alcohol in the first trimester of pregnancy (Zuccolo et al., 2009). The total number of children included in these secondary analyses was 3,114, of whom 176 (5.6%) had mothers with at least one copy of the rare allele.

Single nucleotide polymorphisms were genotyped by KBioscience (http://www.kbioscience.co.uk) using the KASPar chemistry, a competitive allele‐specific PCR system using FRET quencher cassette oligos (http://www.kbioscience.co.uk/ genotyping/genotyping‐chemistry.htm). Blind duplicates and Hardy–Weinberg equilibrium tests were used as quality control checks. Genotyping success rate was above 93.3% and error rate from duplicates was below 0.25% for all SNPs. The top 10 principal components (PCs) that reflect the population's genetic structure were estimated from genome‐wide SNPs genotyped and imputed in ALSPAC children and mothers, using methods described by Price et al. (2006).

### Statistical analyses

We estimated associations (odds ratios and 95% confidence intervals: OR and CI) between the number of child risk alleles (≤2, 3, or ≥4 metabolism impairing alleles), and conduct problem trajectories using multinomial logistic regression. The analysis was stratified by whether or not mothers reported drinking alcohol during pregnancy. Logistic regression was used to examine the association between the maternal genotype and the main outcome identified in analyses of child genotypes.

## Results

Of the 3,544 study children, 72.3% (*N* = 2,561) were classified as having low‐risk conduct problems, 11.5% (*N* = 408) childhood‐limited problems, 8.2% (*N* = 290) adolescence‐onset problems, and 8.1% (*N* = 285) early‐onset‐persistent problems. Table 1 shows that, in crude analyses, maternal drinking in pregnancy was not associated with children's conduct problems, although other maternal characteristics at birth (smoking in pregnancy, depression in pregnancy and low education) were associated with children's conduct problems. Also, mothers who drank moderately in pregnancy were more likely than nondrinking mothers to be older, have a nonmanual social class, and have a higher level of education ‐ demonstrating the potential for bias because of confounding in unadjusted analyses.

**Table 1:** Associations of potential confounders with maternal drinking in pregnancy and child conduct problem trajectories

	Maternal drinking in pregnancy			Child conduct problem trajectory				
	None	Moderate	*p* value	Low risk	Childhood‐limited	Adolescence‐onset	Early‐onset‐persistent	*p* value
	*N* = 1,359	*N* = 2,185		*N* = 2,561	*N* = 408	*N* = 290	*N* = 285	
Mother drank moderately in pregnancy	–	–	–	61.3%	64.5%	62.1%	60.4%	.633^a^
Potential confounders								
Mother smoked in pregnancy	15.0%	13.0%	.099^a^	12.1%	17.4%	14.8%	22.4%	<.001^a^
Mother depressed in pregnancy	8.4%	7.8%	.490^a^	6.8%	8.8%	12.0%	14.3%	<.001^a^
Manual social class	16.4%	12.3%	.001^a^	13.2%	16.3%	14.1%	16.4%	.270^a^
Mother low education	62.9%	47.6%	<.001^a^	52.3%	55.9%	51.4%	63.2%	<.001^a^
Mother's age in years: Mean (SD)	28.5 (4.5)	29.8 (4.3)	<.001^b^	29.4 (4.3)	29.2 (4.7)	29.0 (4.5)	28.9 (4.8)	.001^c^

% = column percent.

a Chi‐squared test.

b *t*‐test.

c Linear regression analysis.

Table 2 shows potential confounding factors by children's genotype score. There was no evidence of association between the genotype score and maternal smoking in pregnancy, maternal depression in pregnancy, maternal social class and maternal education. Although maternal age was associated with children's genotype score, differences across groups were small: mean maternal age was 29.3 for genotype score ≤2, 29.1 for genotype score 3, and 30.0 for genotype score ≥4. Therefore, as expected, children's genotype score represents a relatively unconfounded proxy variable for prenatal alcohol exposure.

**Table 2:** Associations between potential confounders and child genotype score

	Child genotype score (number of risk alleles^a^)			
	≤2	3	≥4	*p* value
	*N* = 1,400	*N* = 1,552	*N* = 592	
Potential confounders				
Mother smoked in pregnancy	13.7%	13.3%	15.4%	.449^b^
Mother depressed in pregnancy	8.4%	7.0%	9.7%	.092^b^
Manual social class	14.6%	12.7%	14.8%	.294^b^
Mother low education	52.7%	54.0%	54.0%	.757^b^
Mother's age in years: Mean (SD)	29.3 (4.5)	29.1 (4.4)	30.0 (4.4)	.016^c^

% = column percent.

a Total number of risk alleles in *ADH1A* rs2866151 rs975833, *ADH1B* rs4147536 and *ADH7* rs284779.

b Chi‐squared test.

c Linear regression analysis.

Table 3 shows the key results of the study: the association between children's genotype scores and their conduct problem trajectories, separately for children whose mothers drank moderately in pregnancy and for children of nondrinking mothers. Among children whose mothers drank moderately, a higher genotype score was associated with increased risk for early‐onset‐persistent conduct problems: OR = 1.29, CI = 1.04–1.60, *p *=* *.020. This was not true for children of nondrinking mothers: OR = 0.94, CI = 0.72–1.25, *p *=* *.688. However, evidence was not strong for modification of the genotype effect by maternal drinking, comparing models with and without an interaction term (*p *=* *.192).

**Table 3:** Relationship between child genotype score and conduct problem trajectories, stratified by maternal drinking during pregnancy

	Genotype score^a^ Among children of nondrinking mothers					Genotype score^a^ Among children of mothers who drank moderately				
	≤2	3	≥4		*p* value	≤2	3	≥4	OR (95% CI)	*p* value
	*N* = 525	*N* = 615	*N* = 219	OR (95% CI)		*N* = 875	*N* = 937	*N* = 373		
Conduct problem trajectory										
Low risk	70.5%	74.8%	73.5%	1.00		73.4%	71.6%	68.9%	1.00	
Childhood‐limited	12.4%	9.6%	9.6%	0.83 (0.64–1.06)	.139	11.8%	12.5%	11.5%	1.03 (0.87–1.24)	.720
Adolescence‐onset	8.8%	7.8%	8.7%	0.95 (0.72–1.26)	.718	8.3%	7.7%	9.4%	1.07 (0.86–1.32)	.547
Early‐onset‐persistent	8.8%	7.8%	8.7%	0.94 (0.72–1.25)	.688	6.5%	8.2%	10.2%	1.29 (1.04–1.60)	.020

% = column percent.

Each OR represents the increased odds per unit increase in genotype score of being in a conduct problem trajectory (early‐onset, childhood‐limited or adolescence‐onset) compared to being in the low‐risk trajectory. Two models were calculated using multinomial logistic regression, one for children of nondrinking mothers, one for children of mothers who drank moderately.

a Total number of risk alleles in *ADH1A* rs2866151 rs975833, *ADH1B* rs4147536 and ADH7 rs28477.

In sensitivity analyses adjusting for maternal age and 10 principal components for population stratification from GWAS (Genome Wide Association Study), results for early‐onset‐persistent conduct problems in children of drinking mothers were unchanged: OR = 1.27, CI = 1.01–1.60, *p *=* *.054. In secondary sensitivity analyses, we ran the same tests, but also included all other perinatal variables (maternal smoking, depression, social class and education). Again, the effect size did not change substantially: OR = 1.26, 0.97–1.62, *p *=* *.078.

We also examined the relationship between children's conduct problems and the maternal genotype *ADH1B* wildtype (vs. rare allele) which is associated with drinking more during pregnancy. This analysis was underpowered due to the low frequency of the rare allele (5.6% minor allele frequency). The results were in the same direction as the results for children's genotype scores, although they were very imprecise given the low statistical power of the test: children with early‐onset‐persistent conduct problems were most likely to have mothers with the wild‐type homozygous genotype (OR = 1.33, CI = 0.71–2.50, *p *=* *.365, using children in the low‐risk conduct group as reference category). Results with adjustment for mother's ancestry‐informative principal components from the GWAS were OR = 1.20, CI = 0.60–2.44, *p *=* *.601.

To examine whether maternal moderate drinking in pregnancy influenced children's conduct problems independently of low child IQ, we reestimated the effects of children's genotype score adjusting for child IQ in a model also including 10 principal components for population stratification and maternal age. Adjustment for IQ did not change the association between children's genotype score and early‐onset‐persistent conduct problems: OR = 1.26, CI = 0.97–1.63, *p *=* *.078, for children of drinking mothers; OR = 0.91, CI = 0.66–1.25, *p *=* *.550 for nondrinking mothers.

## Discussion

The key finding in this population‐based study of 3,500 children is that variation in children's alcohol‐metabolising genes predicted increased risk of early‐onset‐persistent conduct problems among children whose mothers drank moderately during pregnancy, although it did not affect childhood‐limited or adolescence‐onset conduct problems. Causal inference about the effects of prenatal alcohol exposure was strengthened in two ways. First, the genotype variables used as proxies for foetal alcohol exposure were not associated with other maternal characteristics such as education and socioeconomic position. Second, children's genotype scores predicted early‐onset‐persistent conduct problems *only among children whose mothers drank in pregnancy*. Among children of nondrinking mothers, children's conduct problems were not predicted by their genotype score.

This study suggests that moderate drinking in pregnancy increases risk for children's conduct problems, corroborating the suggestion that foetal alcohol syndrome represents just the ‘tip of a morbidity iceberg’ (Gray, Mukherjee, & Rutter, 2009). The results are consistent with studies showing adverse effects of moderate alcohol exposure on brain development (Ikonomidou et al., 2000; Mattson et al., 2001) and two previous studies of children's conduct problems (D'Onofrio et al., 2007; Sood et al., 2001). Only one prior study of child behaviour used a quasi‐experimental design (sibling‐comparisons) to adjust for unmeasured confounders (D'Onofrio et al., 2007). In that study, there was a dose–response relationship between maternal drinking levels in pregnancy and children's conduct problems, but no effects were found for children's hyperactivity. The current study suggests further possible specificity of effects: foetal alcohol exposure particularly influences the development of early‐onset‐persistent conduct problems, rather than childhood‐limited or adolescence‐onset problems. This finding is consistent with Moffitt's theory that different developmental trajectories of conduct problems have different causes, and that health factors altering neurological functioning are particularly relevant to early‐onset‐persistent problems (Moffitt, 1993; Moffitt & Caspi, 2001). It is important to consider that trajectory groups provide approximations to children's behavioural development, and that the early‐onset persistent trajectory might represent the extreme on a severity continuum of conduct problems, rather than a qualitatively different group (Fairchild, van Goozen, Calder, & Goodyer, 2013).

Several prior studies reported no adverse effects of maternal light‐moderate drinking on children's conduct problems (Kelly et al., 2009, 2010; Larkby et al., 2011; O'Leary et al., 2010; Robinson et al., 2010; Skogerbø et al., 2013). If replicated, the different conclusion of the current study might be explained by the use of Mendelian randomisation to reduce confounding, or the use of a more refined outcome based on longitudinal trajectories, which separated onset and persistence of children's conduct problems.

Effects of prenatal alcohol exposure on children's conduct problems were not accounted for by children's IQ in this study. This suggests that there are multiple consequences of moderate drinking for children, as effects on IQ (Lewis et al., 2012) as well as school performance (Zuccolo et al., 2013) have been documented previously, and also that effects on children's conduct problems are more likely to be explained by specific neuropsychological mechanisms related to understanding and regulating emotion and behaviour (Raine, 2002), rather than general cognition. In particular, foetal alcohol exposure might influence children's executive functioning via effects on the frontal lobes of the brain, the caudate in the basal ganglia, and the corpus callosum, independently of its effects on overall intelligence (Kodituwakku, Kalberg, & May, 2001; Mattson, Crocker, & Nguyen, 2011; Mattson et al., 2001). Deficits in executive functioning are associated with conduct problems (Ogilvie, Stewart, Chan, & Shum, 2011) because they relate to children's ability to anticipate consequences of behaviour, levels of self‐control and emotional responses to rewards and punishments. Hence, foetal alcohol exposure might increase risk for children's conduct problems via adverse effects on their executive functioning skills. According to Moffitt's (1993) theory, these effects are likely to be observed early in life, and accumulate via stressful social interactions to produce problem behaviours that persist into adolescence and beyond.

### Limitations

Two limitations arose because it is not possible to measure foetal blood‐alcohol levels. First, this study assumed that variants in children's alcohol‐metabolising genes caused different foetal blood‐alcohol levels, but that could not be proved, and it is not known to what extent these genetic variants affect foetal alcohol metabolism. However, the fact that the genetic variants were associated with child outcomes *only if mothers drank in pregnancy* in this study, paralleling the findings of Lewis et al. (2012), supports the assumption that these genetic variants do affect foetal alcohol metabolism. Second, without measures of foetal blood‐alcohol levels, the current study could only answer the qualitative question ‘is maternal moderate drinking related to risk for children?’ – it was not possible to estimate the strength of the effect. As such, we could not compare effects across different levels of maternal drinking to determine whether a dose–response relationship exists, or whether heavy drinking is particularly harmful for children, as suggested by other studies (D'Onofrio et al., 2007). Also, although the Mendelian randomisation design helped improve causal inference, it was not possible to test the specific neuropsychological mechanisms involved linking foetal alcohol exposure and children's behaviour. Another limitation is that a considerable number of children could not be included in the analyses because of missing data. As a result, the analyses had lower statistical power and findings may not generalise to children who were excluded because of missing data, who tend to come from more deprived social backgrounds. However, the main analysis of the effect of genotype on behaviour should not be affected by missing data, as genotype is not related to having missing data. Statistical power was low for detecting an interaction between maternal drinking in pregnancy, genotype score and children's outcomes. The weak evidence for an interaction reduces confidence in the different genetic associations between children of drinking and nondrinking mothers. Nonetheless, it does not affect the main finding of an association in children of moderate alcohol drinking mothers. Another limitation that should be considered is that the last assessment of conduct problems included in the study was at 13 years, and some individuals in the low‐risk group might develop conduct problems later in adolescence; more years of outcome data could change trajectory group membership. Finally, the use of maternal self‐reports on alcohol consumption in pregnancy might mean that alcohol use was underestimated, and the observed results might reflect effects of higher than moderate drinking levels.

## Conclusion

The key implication of this study is that moderate alcohol drinking in pregnancy represents a possible preventable cause of persistent child conduct problems and, in the absence of definitive evidence about safe levels of alcohol use, conservative recommendations against drinking alcohol during pregnancy are appropriate. As well as replications of these findings, future research should assess when in pregnancy it is most critical to avoid drinking and how effects on children vary with different levels and patterns of drinking. In particular, it is important to establish whether low levels of alcohol use are safe, or whether complete abstinence is necessary.

**Key points**

- Evidence is inconsistent about possible effects of moderate drinking in pregnancy on children's behavioural development; this may be because of difficulties with testing causal effects in observational studies.
- This study used a Mendelian randomisation study design to improve causal inference about effects of foetal alcohol exposure on children's conduct problems.
- Genetic variants in children associated with alcohol metabolism predicted children's early‐onset‐persistent conduct problems among women who drank during pregnancy, but not among those who abstained during pregnancy.
- This quasi‐experimental study suggests that moderate alcohol drinking in pregnancy increases risk for children's early‐onset‐persistent conduct problems, but not childhood‐limited or adolescence‐onset conduct problems.
